# A rare presentation of Pulmonary Lymphangitic Carcinomatosis in cancer of lip: case report

**DOI:** 10.1186/1477-7819-9-77

**Published:** 2011-07-14

**Authors:** Sajith Babu, Satheeshan B, Geetha M, Surij Salih

**Affiliations:** 1 Department of Surgical Oncology, Malabar Cancer Centre, Thalassery, Kerala; 2 Department of Radiation Oncology, Malabar Cancer Centre, Thalassery, Kerala

## Abstract

Squamous cell carcinoma of lip is a common malignancy in Indian subcontinent. Metastatic spread is infrequent. Although advanced tumours spread to lymph nodes in the neck, it does not typically present with lung metastasis or with lymphangitic carcinomatosis. We describe a patient who developed cough and increasing dyspnoea while on treatment for carcinoma of lip. Chest x-ray and computed tomography were consistent with lymphangitic carcinomatosis. Lymphangitic carcinomatosis occurs with many different primary tumours and can rarely occur in oral cancers. This is the first report from carcinoma of lip.

## Background

The common site of metastasis from most of the solid malignancies is lung. They usually appear as nodular lesions in radiologic images. In some patients, metastasis presents with interstitial spread and it is referred to as Pulmonary Lymphangitic Carcinomatosis (PLC). Head and neck cancers very rarely have lung metastasis in the form of PLC. Oropharyngeal and hypopharyngeal cancers have been reported to have such type of metastasis [1]. Cancer of lip is a common malignancy in Indian subcontinent mainly due to tobacco chewing and that these cancers are detected in early stages due to its visible location, a spread to lung is rare and they are of typical nodular metastases. PLC has not been reported till date from lip cancers in English literature. Here we report a case of PLC arising from cancer of the lower lip.

## Case Presentation

**Figure 1:**
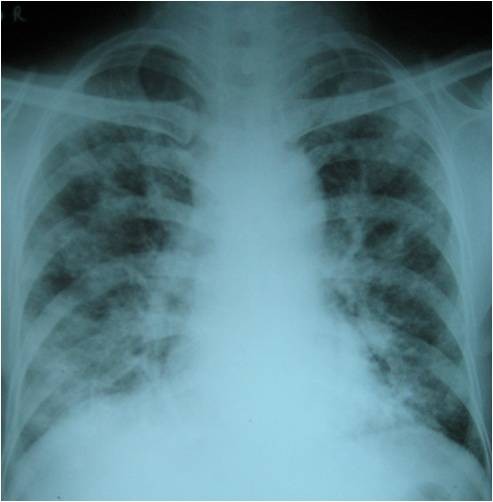
**CXR: Chest Radiograph showing septal lines**.

**Figure 2:**
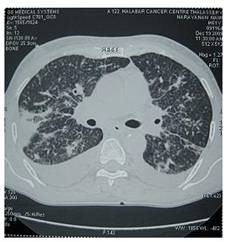
**CT Scan: CT scan of thorax showing diffuse and bilateral findings**.

60 year old gentleman with no co morbid illness, presented with a squamous cell carcinoma of lower lip. After evaluation, this was staged as T4 N2a M0, stage IV and was moderately differentiated squamous cell carcinoma. The X-ray of the chest was within normal limits. Wide excision of the lesion and reconstruction with a deltopectoral flap and a radical neck dissection on ipsilateral side was done. Postoperative histopathology was moderately differentiated squamous cell carcinoma (pT4 N2a). After 4 weeks, post operative adjuvant concurrent chemo radiation was started with Cisplatin and radiotherapy in 2 Gy per fraction. While on radiotherapy, the patient developed severe dyspnoea of acute onset. There was no history of similar episode in the past and he was not a known patient of chronic obstructive pulmonary disease. He was afebrile and there was no cough or expectoration. Basic haematological study revealed normal haemogram. Clinically he was dyspnoeic, tachypnoeic and with tachycardia. On auscultation of the chest, there was scattered crackles and occasional ronchi. Air entry was equal on both sides. He was put on symptomatic care in the form of bronchodilators, antibiotics and nasal oxygen. Possibilities considered were acute bronchopneumonia and PLC. Chest radiograph revealed interstitial linear pattern from the hilum to the outer lung fields (Figure 1) and Kerley's B lines in both lungs suggesting PLC. A computerized tomography was taken which showed nodular septal thickening and it strongly suggested the diagnosis of PLC (Figure 2). Patient was given further courses of chemotherapy with Cisplatin, but with no improvement. The patient succumbed to disease on eighteenth day after the start of pulmonary symptoms.

## Discussion

Lung metastasis from malignant tumours usually present as nodular lesions and rarely as Pulmonary Lymphangitic Carcinomatosis (PLC). PLC is characterised by diffuse spread of malignancy in the lung, causing inflammation of the lymph vessels. The first reported case of PLC was by Gabriel Andral in 1829 [2]. The diffusely infiltrating pattern of metastasis as seen in PLC occurs in 6-8% of lung metastases [3]. 80% of them are from adenocarcinomas. The common sites of primary from which PLC occurs are cancers of breast, bronchus, and stomach [4,5]. The other described sites with PLC are cancers from colon, pancreas, kidney, cervix, thyroid, larynx and hypopharynx [6-8].

The cancers of head and neck rarely show this type of metastasis. The exact reason is unknown. The described sites in head and neck region are larynx, hypopharynx and thyroid. Metastasis to lymph nodes from advanced cancers of lip is seen in about 44%. Metastasis to lung is reported to be very low. There is no available report suggesting a PLC from oral cancers. PLC as metastatic feature as seen in the case described in this manuscript is an extremely rare presentation.

The pathophysiology is that the tumours spread by haematogenous route to the lung and then through the lymphatics within the lung. The lymphatics in the lung are seen in the peribronchovascular, centrilobular, interlobular and sub pleural regions. The tumour obstructs these lymphatic channels. The dilated lymphatic vessels due to oedema fluid, tumour secretion and the desmoplastic reaction by the tumour cells, produces interstitial thickening which is seen as streaks in imaging studies. The nodular pattern is due to the spread of tumour into the lung parenchyma as seen in usual lung metastases.

The clinical features of PLC are dyspnoea and nonproductive cough with crepitations and without features of consolidation. Chest X-ray shows septal lines (Kerley A and B lines). The differential diagnosis is interstitial lung disease, primary malignancy in the lung, pulmonary sarcoidosis and hypersensitivity pneumonitis. HRCT is the modality of choice for confirmation of the diagnosis. The findings in CT scan are - thickening of interlobular septa, fissures and bronchovascular bundles. These findings may be seen as limited or diffuse and may involve unilateral or bilateral lungs. The radiologic picture may be symmetric or asymmetric in both lungs. The other findings are nodularity in pleura and ground glass opacity [9]. The possibility of interstitial lung disease is to be considered and ruled out. Prakash P et al described the use of PET/CT in diagnosing PLC. In a study of 35, they found that PET/CT has high specificity in detection of pulmonary lymphangitic carcinomatosis [10].

Histopathological examinations show interstitial oedema and fibrosis along with malignant cells and are found usually on postmortem biopsy. Since the radiological finding in a patient with malignant disease elsewhere is suggestive, a biopsy of the lung is not mandatory.

PLC often presents in the late stages of malignancy and it indicates poor prognosis. The treatment option in PLC is with chemotherapy. Cisplatin have been found to be effective [11].

## Conclusion

Pulmonary Lymphangitic Carcinomatosis may also occur rarely in patients with oral cancers as seen in our patient and its prognosis is very poor even with treatment with chemotherapy.

## Consent

Written informed consent was obtained from the patient for publication of this case report and accompanying images. A copy of the written consent is available for review by the Editor-in-Chief of this journal.

## Competing interests

The authors declare that they have no competing interests.

## Authors' contributions

SB prepared the manuscript and the literature search, GM reviewed and edited the manuscript, ST corrected and revised the manuscript, SS: reviewed the manuscript. All authors read and approved the final manuscript.
